# Return sweeps in reading: Processing implications of undersweep-fixations

**DOI:** 10.3758/s13423-019-01636-3

**Published:** 2019-07-19

**Authors:** Timothy J. Slattery, Adam J. Parker

**Affiliations:** 1 Department of Psychology, Faculty of Science & Technology, Bournemouth University, P104c, Poole House, Talbot Campus, Fern Barrow, Poole, Dorset BH12 5BB UK; 2 University of Oxford, Oxford, UK

**Keywords:** Undersweep-fixations, Reading, Eye movements, Return sweeps, Inhibition of return

## Abstract

Models of eye-movement control during reading focus on reading single lines of text. However, with multiline texts, return sweeps, which bring fixation from the end of one line to the beginning of the next, occur regularly and influence ~20% of all reading fixations. Our understanding of return sweeps is still limited. One common feature of return sweeps is the prevalence of oculomotor errors. Return sweeps, often initially undershoot the start of the line. Corrective saccades then bring fixation closer to the line start. The fixation occurring between the undershoot and the corrective saccade (undersweep-fixation) has important theoretical implications for the serial nature of lexical processing during reading, as they occur on words ahead of the intended attentional target. Furthermore, since the attentional target of a return sweep will lie far outside the parafovea during the prior fixation, it cannot be lexically preprocessed during this prior fixation. We explore the implications of undersweep-fixations for ongoing processing and models of eye movements during reading by analysing two existing eye-movement data sets of multiline reading.

## Introduction

As we read our eyes move from one location on the page to another in fast jumps, called saccades. Return sweeps are saccades made at the end of a line of text in order to fixate the subsequent line, and often undershoot line initial words (Hofmeister, Heller, & Radach, 1999; Parker, Kirkby, & Slattery, 2017). These undershoots are followed by a corrective saccade that brings fixation closer to the start of the line. The short pauses between the return sweep and the corrective saccade are termed undersweep-fixations (Parker et al., 2017). They are an interesting test case for serial attention shift models of eye-movement control during reading as they occur on words ahead of the serial order and attentional targeting. A tacit assumption in the field of eye-movement reading research has been that undersweep-fixations are simply the result of oculomotor error, reflecting little to no influence of ongoing linguistic processing. Indeed, multiline reading studies typically exclude the fixations around return sweeps from analysis (see Table 1). As such, it remains unclear if and how the glimpse of a word afforded to readers during undersweep-fixations influences subsequent reading. These issues are the focus of the current study.

**Table 1:** Examples of authors choosing data trimming procedures that exclude return-sweep saccades and fixations from analysis

Authors	Quote
Hand et al. (2010)	“Data were additionally eliminated if *. . .* the fixation on the target was either the first or last fixation on a line.”
Hand, O’Donnell, and Sereno (2012)	“Data were additionally eliminated if *. . .* the fixation on the target was either the first or last fixation on a line.”
Kuperman et al. (2010)	“. *. .* we excluded fixations that landed on the first or the last word of a line or of a sentence for compatibility with other data sets and to avoid the potential influence of the eye movement behaviour at line breaks.”
Miellet, Sparrow, and Sereno (2007)	“In accordance with E-Z Reader 7, the first and last words of each line of text were excluded from the simulation.”
Pynte and Kennedy (2006)	“The first word in each line was thus excluded from the data set”
Rayner, Slattery, Drieghe, and Liversedge (2011)	“Return sweeps from the first to the second sentence that landed on or beyond the target word were also excluded from analysis.”
Whitford and Titone (2012)	“Following prior work . *. .* words at the beginning and end of every line of text were removed from analyses.”
Whitford and Titone (2014)	“We excluded words at the beginning and end of every line of text”
Henderson et al. (2013)	“. *. .* fixations could not be followed within 700 ms by a return sweep.”

### Return sweeps

Like all saccades, return sweeps are subject to saccadic range error and tend to undershoot their target by about 10% (Becker, 1972). Frequently, return sweeps fall short of the start of a new line and are followed by a corrective saccade that brings fixation closer to the left margin (Hofmeister et al., 1999; Parker et al., 2017; Parker, Nikolova, Slattery, Liversedge, & Kirkby, 2019; Parker, Slattery, & Kirkby, 2019; ; Slattery & Vasilev, 2019). In such cases, the intervening undersweep-fixation tends to be shorter than typical reading fixations (138–176 ms; Heller, 1982; Parker, Kirkby et al., 2019). The short duration of undersweep-fixations results from oculomotor error. Corrective saccades are quickly initiated based on retinal feedback that the eyes landed far from their intended target (Becker, 1976; Hofmeister et al., 1999).

Undersweep-fixations can also complicate data analysis for eye-movement studies of reading. Many dependent measures used in this field are contingent on “first-pass reading.” First-fixation duration, single-fixation duration, and gaze duration for a given word are only defined if a fixation enters the word from an earlier region of text prior to a fixation occurring on a later region of text. So, in cases where a word is skipped and then regressed back to, these fixations would not be counted toward first-pass reading time measures. Therefore, undersweep-fixations prematurely terminate first-pass reading for all the words on the line that come before it. For this reason, multiline eye-movement studies typically remove the first fixation on a line, or at very least the undersweep-fixation (see Table 1). For instance, Hand, Miellet, O’Donnell, and Sereno (2010) removed data if the fixation was either the first or last fixation on a line, whereas Kuperman, Dambacher, Nuthmann, and Kliegl (2010) excluded fixations that landed on the first or last word of a line to avoid the influence of return sweeps. Such decisions may have implications for those interested in reading times and word skipping, as the information acquired during these fixations may influence later eye-movement behaviour. For instance, when line initial fixations are removed, a target word receiving an undersweep-fixation which is subsequently skipped may wrongly be viewed as having been processed without direct inspection. To avoid such an issue, as with Rayner et al. (2011), trials in which return sweeps landed on or beyond the target may be excluded from an analysis. However, this decision may lead to the unnecessary exclusion of data. What is needed is a better understanding of how undersweep-fixations may be involved in reading processes.

It remains unclear if lexical information of the fixated word is acquired during an undersweep-fixation. Given the tacit belief that undersweep-fixations are the result of low-level oculomotor error correction, it is often assumed that useful lexical information is not obtained during an undersweep-fixation. However, this assumption has yet to be empirically evaluated. If true, then during the left-to-right reading pass of the line, words which earlier received undersweep-fixations should have similar skipping rates and gaze durations as words which did not receive undersweep-fixations.

### Time course of lexical processing

Undersweep-fixation durations are roughly half the duration of standard reading fixation durations (~130 ms vs. ~250 ms). While cognitive control theories of reading assert that fixation durations are strongly influenced by linguistic processing (Rayner, 1998, 2009), according to the strategy tactics (O’Regan & Levy-Schoen, 1987), race model (McConkie & Dyre, 2000), and minimal control model (Suppes, 1990), linguistic processing has no role or a very limited role on reading fixation durations. McConkie and Dyre (2000) assert that there exists an early set of saccades that are initiated without any influence from the stimulus properties located at fixation (see also Yang & McConkie, 2001).

More recent research found evidence for direct cognitive control of reading fixations (Dambacher, Slattery, Yang, Kliegl, & Rayner, 2013). Dambacher et al. (2013) used a gaze contingent display technique to delay (with letter masks) the appearance of words during reading. Across two experiments they found that the extent of the delay translated into a nearly equivalent increase in fixation durations. However, the authors noted that there was a subpopulation of early saccades that were triggered from nonoptimal fixation locations which were an exception to this rule.

What is the earliest point at which higher level cognitive processes related to lexical analysis can affect the duration of reading fixations? This question has been recently addressed with the use of survival analysis (Reingold, Reichle, Glaholt, & Sheridan, 2012; Reingold & Sheridan, 2018). Based on this approach, the earliest influence of lexical properties on fixation durations occurs in the range of 110–150 ms after the start of fixation. Therefore, the undersweep-fixation durations are at the edge of where it is possible for lexical effects to be detected.

Failing to reliably detect influences of lexical variables on the durations of undersweep-fixations would not imply the absence of lexical processing during these brief fixations. Indeed, research using the disappearing text paradigm (Rayner, Liversedge, White, & Vergilino-Perez, 2003), in which text is masked or disappears during a fixation, has shown that words can be encoded in as little as 50–60 ms. However, in the disappearing text paradigm, the eyes remain fixated on the word location even after it has disappeared, with this duration being modulated by word frequency (Blythe, Liversedge, Joseph, White, & Rayner, 2009; Liversedge et al., 2004; Rayner, Liversedge, & White, 2006). Furthermore, Rayner et al. (2006) show that initial encoding of the word in parafoveal preview is vital in combination with the 60-ms foveal presentation. Such a parafoveal preview wouldn’t be available for the undersweep-fixations discussed here.^1^

Therefore, undersweep-fixations are theoretically long enough to encode the words that they land on. However, the signal to initiate the next saccade may be occurring with little (or no) information from lexical processing.

### Eye-movement models

Models of eye-movement control during reading fit data from single-sentence reading studies which are devoid of return sweeps. Currently, we know of no such model that includes a mechanism for return-sweep saccades. However, aspects of existing models may, in principle, be able to account for aspects of return sweeps and undersweep-fixations via oculomotor control mechanisms. For instance, within E-Z Reader (Reichle, Pollatsek, Fisher, & Rayner, 1998; Reichle, Rayner, & Pollatsek, 2012), not all saccades land on their targeted word (Drieghe, Rayner, & Pollatsek, 2008) due in part to simulated saccadic range error. According to E-Z Reader, the probability of immediately programming a corrective saccade increases as the distance between the actual and intended fixation locations increase. This mechanism may explain the short undersweep-fixations followed by corrective regressions. Note that while E-Z Reader simulates error in the movement of the eyes, it assumes there is no error in the serial movement of attention. Therefore, during an undersweep-fixation, attention for word processing, within the model, would be allocated to the first word on the line rather than the fixated word, and no lexical information about the fixated word should be acquired. If words receiving an undersweep-fixation are lexically processed to some degree during that fixation, this would be more consistent with distributed lexical processing models such as SWIFT (Engbert, Nuthmann, Richter, & Kliegl, 2005; Schad & Engbert, 2012).

### Inhibition of return

While eye movements during reading are typically under linguistic control, oculomotor effects such as inhibition of return (IoR) have been observed (Eskenazi & Folk, 2017; Henderson, Luke, Schmidt, & Richards, 2013; Rayner, Juhasz, Ashby, & Clifton, 2003). Inhibition of return is the finding that it takes longer to send attention back to a recently attended location (Posner & Cohen, 1984; see Klein, 2000, for a comprehensive review). Inhibition of return effects during reading are characterised by increased fixation durations prior to saccades that immediately return the eyes to a previously attended word. Most of the reading research examining IoR has focussed on regressive saccades back to previously fixated or skipped words. However, Rayner et al. (2003) also examine the effect of IoR on forward saccades following regressions. They reported that the fixation durations prior to such forward saccades were longer if they returned to a word that had been fixated on the immediately prior fixation (forward return saccade) than if they did not return to this word (forward nonreturn saccade). Given that the majority of undersweep-fixations occur on the second word of a line, it is likely that IoR will influence a substantial portion of the subsequent fixations (i.e., those which follow the corrective leftward saccade). Therefore, a secondary purpose of the current study is to assess the extent to which IoR plays a role in fixation durations following undersweep-fixations. It may be possible that during an undersweep-fixation, attention is actually located at the target location of the upcoming corrective saccade. If this was the case, then we would expect a lack of IoR in cases when readers return immediately to the location of the undersweep-fixation.

### Current study

To better understand the influence of undersweep-fixations during reading, we present analyses of two existing eye-movement data sets of multiline reading. For each data set, we use linear mixed models to explore three main questions:

1. Are the durations of undersweep-fixations influenced by the lexical characteristics of the words they land on?
2. Do words receiving undersweep-fixations show evidence of earlier processing during the subsequent reading pass of the line?
3. To what extent does IoR influence the fixations immediately following undersweep-fixations?

## Method

**Fig. 1:**
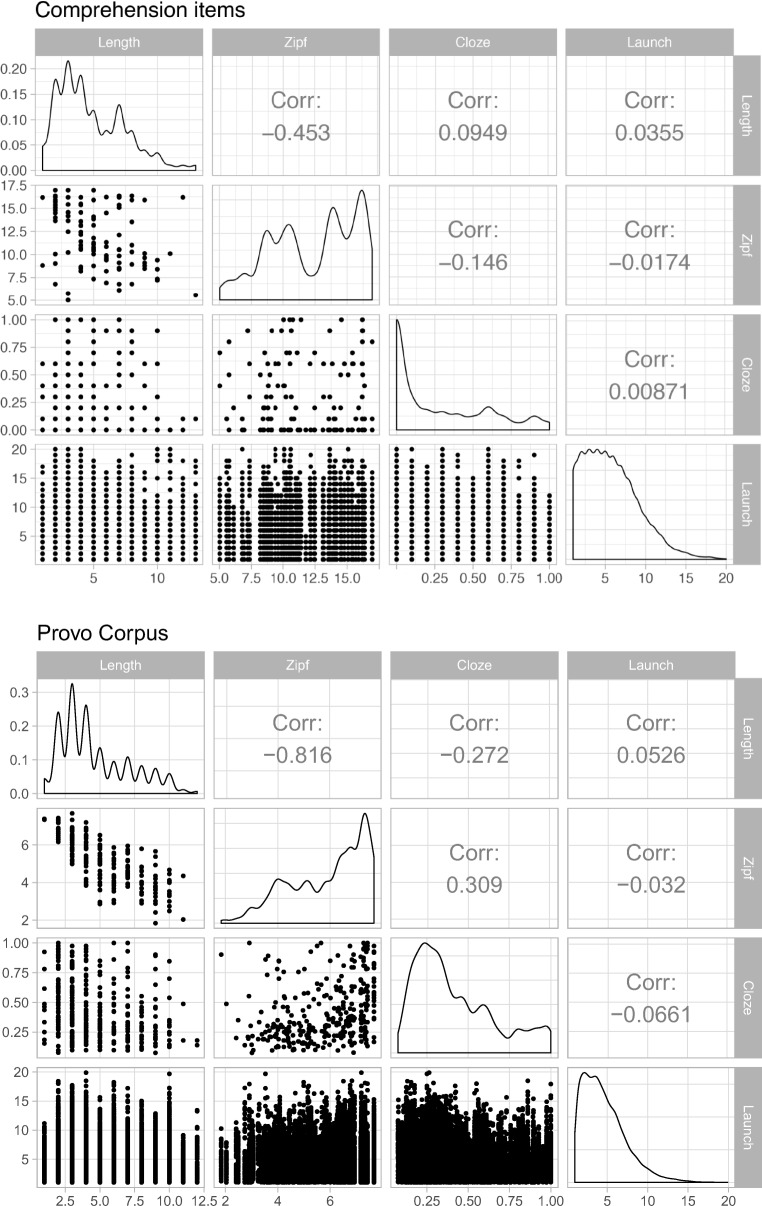
Correlation coefficients, scatterplots, and distributions for variables in the comprehension (top panel) and Provo Corpus (bottom panel)

To answer these questions, we performed novel analyses of two existing multiline reading data sets. Each set contained information about the frequency, length, and cloze predictability of the words in each passage. Average word frequency and cloze norming information is shown in Fig. 1. All data were collected with an SR Research EyeLink 1000 tracker.

**Fig. 2:**
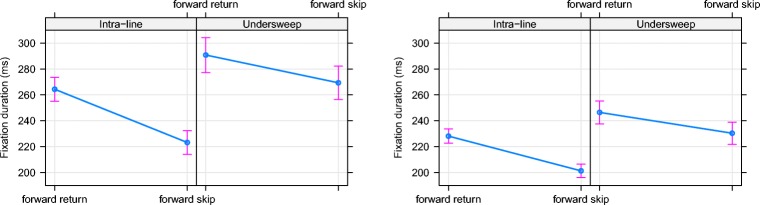
Fixed effects estimates from linear mixed-effects models for fixation duration prior to a forward return saccade or a forward skips of a previously fixated word for intraline and undersweep-fixations. 95% confidence intervals are presented around the mean

### Data sets

#### Comprehension items

In total, 143 participants read three multiline passages to assess reading comprehension. Forty-eight of these participants were from Bournemouth University (UK), and the remaining 95 were from the University of South Alabama (USA). Each passage was 120 words in length with an average word length of 5.07 characters (for a full description, see Slattery & Yates, 2018).

#### Provo Corpus

The Provo Corpus (Luke & Christianson, 2018) consists of 55 short multiline passages, with an average of 50 words (range: 39–62), which were silently read by 84 participants (for a full description see Luke & Christianson, 2018).

### Dependent measures

To address our questions, we analyse three eye-movement measures. The first is the duration of undersweep-fixations. We define an undersweep-fixation as the first fixation following a return sweep given that the next saccade moves to the left. The other measures are “first pass” reading measures: Skipping rate and gaze duration. To calculate these measures, we ignore undersweep-fixations. However, words were dummy coded as having received an undersweep or not for analysis. If words are not processed when an undersweep-fixation lands on them, then words receiving one should be just as likely to be skipped as words that do not receive one. Similarly, there should be no difference in gaze durations based on whether or not a word received an undersweep-fixation.

## Results

We computed linear mixed-effect models (LMMs) using the *lmer* function from package lme4 (Version 1.1–18; Bates et al., 2018) in R (R Core Team, 2018). Values for undersweep-fixation and gaze duration were log transformed. We report regression coefficients (*b*), standard errors (*SE*), and *t*-values. We consider |*t*| > 1.96 as statistically significant (Baayen, Davidson, & Bates, 2008). For skipping likelihood, we used generalized linear mixed-effect models (*glmer* function from package lme4) and report the Wald *z*. Initially, all models adopted a full random structure for participants and items, with random intercepts and slopes (Barr, Levy, Scheepers, & Tily, 2013). If models failed to converge, we removed random effects parameters to reduce overfitting so long as these removals did not reduce model fit (Bates, Kliegl, Vasishth, & Baayen, 2015). All numerical variables were centred prior to analysis. Regression coefficients are shown in Table 3. Critically, only Words 2–4 on each line entered our analyses as less than 5% of return sweeps landed beyond Word 4 in these samples. Fixations less than 80 ms which were within one character of a temporally adjacent fixation were merged with that fixation. All other fixations less than 80 ms were excluded from analysis, as were fixations greater than 800 ms. Return-sweep saccades that traversed fewer than 25 characters were excluded from analysis. This led to the removal of 9.84% of return sweeps from the comprehension items and 4.20% from the Provo Corpus. Undersweep-fixations were followed by more than one leftward saccade in 8.36% of cases in the Provo Corpus and 11.98% in the comprehension items. In these multiple corrective saccade cases, only the first fixation was coded as an undersweep. See Table 2 for more information about data-entering analysis.

**Table 2:** Mean durations and skipping rates for words entering analysis from comprehension items and the Provo Corpus

Corpus	Observations	Undersweep-fixations	Undersweep duration	Gaze duration	Skipping rates
Comprehension items	10,051	1,076	151.6 (54.30)	302.3 (165.61)	36.5 (12.48)
Provo Corpus	10,047	2,316	126.4 (33.14)	261.1 (128.72)	47.7 (13.50)

Counts are shown for observations and undersweep-fixations. Means are shown for undersweep-fixation duration, gaze duration, and skipping rate. Standard deviations are shown in parentheses. Observations refer to the amount of words entering analysis across all subjects and items

### Undersweep-fixation durations

To assess whether the duration of the undersweep-fixation was influenced by lexical variables, we fit LMMs to log-transformed undersweep-fixation duration data. While the final LMMs yielded different random effects structures, the fixed effects indicated that undersweep-fixation durations were uninfluenced by word length, frequency, or predictability of the word they landed on.

### Word skipping

The final models for both data sets had the same random effects structure. Analysis of each data set indicated that skipping increased with increasing word frequency and predictability, and decreased with increasing length and for launch sites further away. Additionally, in both data sets, word skipping was more likely if a word had received an undersweep-fixation prior to the left-to-right pass.

### Gaze duration

While the final LMMs fit to the two data sets yielded different random effects structures (see Table 3), the fixed effects for the two models were very similar. Recall that gaze was calculated for words based on the left-to-right reading of the line and excluded undersweep-fixations. Gaze durations increased with decreasing word frequency, increasing word length, and increasingly distant launch sites. Furthermore, the gaze durations on words that received an undersweep-fixation were significantly shorter than on words which did not receive an undersweep-fixation.

**Table 3:** LMM coefficients for undersweep-fixation duration and skipping and gaze duration analysis during subsequent reading.

Undersweep-fixation duration						
	Comprehension items			Provo Corpus		
Random effects	*(1 + Cloze | Subject) + (1 + Cloze | Item)*			*(1 | Subject) + (1 + Cloze + Frequency | Item)*		
Predictor	*b*	*SE*	*t*	*b*	*SE*	*t*
Intercept	2.171	0.008	286.51	2.095	0.006	**323.26**
Length	−0.001	0.001	−0.75	−0.002	0.001	−1.73
Frequency	−0.002	0.001	−1.53	0.000	0.003	0.029
Cloze	0.010	0.024	0.42	−0.000	0.012	−0.011
Word skipping						
	Comprehension items			Provo Corpus		
Random effects	*(1 | Subject) + (1 | Item)*					
Predictor	*b*	*SE*	*z*	*b*	*SE*	*z*
Intercept	−1.162	0.090	**−12.85**	−0.415	0.114	**−3.64**
US	0.850	0.090	**9.45**	0.478	0.060	**7.92**
Length	−0.435	0.025	**−17.59**	−0.442	0.017	**−26.68**
Frequency	0.428	0.035	**12.27**	0.315	0.026	**12.02**
Cloze	0.303	0.088	**3.43**	0.362	0.095	**3.81**
LS	−0.289	0.010	**−29.26**	−0.258	0.008	**−30.94**
Gaze duration						
	Comprehension items			Provo Corpus		
Random effects	*(1 + US + Cloze | Subject) + (1 + Cloze | Item)*			*(1 + US + Cloze | Subject) + (1 | Item)*		
Predictor	*b*	*SE*	*t*	*b*	*SE*	*t*
Intercept	5.545	0.029	**191.34**	2.348	0.008	**294.46**
US	−0.054	0.023	**−2.33**	−0.014	0.005	**−2.60**
Length	0.022	0.003	**6.78**	0.011	0.001	**8.59**
Frequency	−0.030	0.006	**−5.12**	−0.011	0.002	**−4.86**
Cloze	−0.083	0.057	−1.45	−0.021	0.015	−1.40
LS	0.013	0.002	**7.93**	0.008	0.001	**12.75**

Significant *t* and *z* values (|*t/z*| >= 1.96) are printed in bold. All models had the same fixed effects structure which only included additive effects of the predictors

*US* undersweep, *LS* launch site

### Inhibition of return

Given the penalty associated with returning to a previous attended location, the increased skipping rates and shorter gaze durations on words previously fixated during an undersweep-fixation may be related to IoR. To examine such a possibility, we systematically examined the two corpora for evidence of IoR.

First, we assessed the extent to which IoR effects exist for intraline fixations within these data sets. To achieve this, we compared the fixation duration prior to a rightwards saccade to a new location to that of a rightwards saccade to a word that had been fixated on the immediately prior fixation. We focused only on the rightward saccades for comparison, as the critical return saccades to words which received an undersweep-fixation must be rightward saccades. For these analyses, we included saccade length as a control variable. Following Rayner et al. (2003), we limited saccades to be 3–12-characters in length (see Table 4 IL replication for descriptive values). The model, fit to log-transformed fixation duration, indicated that fixation durations were longer prior to a saccade to a previously fixated word in both corpora (see Table 5). Therefore, there is evidence within both data sets for IoR with intraline saccades.^2^

**Table 4:** Number of observations, average preceding fixation duration and incoming saccade length

	Comprehension Items					
	IL Replication		Undersweep Skip Comparison			
	FNR	FR	FR	FS	FR	FS
			IL	IL	US	US
N	22,127	2,447	868	968	409	433
PFD	251.0 (102.5)	265.0 (111.6)	266.8 (125.3)	218.9 (91.8)	289.1 (137.8)	260.1 (122.1)
Saccade length	7.7 (2.4)	6.3 (2.5)	6.3 (2.5)	5.7 (2.3)	5.8 (2.2)	5.1 (1.8)
	Provo Corpus					
N	70,750	5,874	977	1,453	607	851
PFD	218.0 (82.0)	230.5 (87.8)	225.9 (85.8)	195.0 (80.1)	246.4 (93.7)	225.6 (81.9)
Saccade length	7.5 (2.4)	6.7 (2.3)	6.6 (2.3)	6.3 (2.3)	6.9 (2.1)	6.4 (2.2)

*Note.* PFD: preceding fixation duration (ms); FNR: forward non-return; FR: forward return; IL: intra-line; US: undersweep-fixation; SL: saccade length; FS: forward skip

**Table 5:** LMM coefficients for inhibition of return (IoR) analyses

Replication (forward nonreturn saccades vs. forward return saccades)						
	Comprehension items			Provo Corpus		
Random effects	*(1 | Subject) + (1 | Item)*			*(1 | Subject)*		
Predictor	*b*	*SE*	*t*	*b*	*SE*	*t*
Intercept	2.363	0.005	**442.47**	2.307	0.005	**438.79**
FR	0.029	0.003	**8.96**	0.031	0.002	**16.57**
SL	0.010	4.05e^-4^	**25.00**	0.010	2.17^e-4^	**48.14**
Forward return saccades vs. forward skip saccades						
	Comprehension items			Provo Corpus		
Random effects	*(1 | Subject) + (1 | Item)*			*(1 + US | Subject) + (1| Item)*		
Predictor	*b*	*SE*	*t*	*b*	*SE*	*t*
Intercept	2.387	0.007	**351.51**	2.333	0.005	**427.96**
US	0.042	0.009	**4.68**	0.033	0.007	**5.10**
FS	−0.069	0.005	**−14.37**	−0.060	0.003	**−21.98**
SL	0.007	0.001	**7.26**	0.010	0.001	**20.42**
US × FS	0.035	0.012	**2.91**	0.033	0.006	**5.55**

Significant *t* values (|*t*| > = 1.96) are printed in bold, *FR* forward return, *US* undersweep-fixation, *SL* saccade length, *FS* forward skip

Next, we explored the possibility of IoR influencing the fixation following an undersweep-fixation. There can be no forward non-return fixations (as defined in Rayner et al., 2003) with undersweep cases. Therefore, we compared the fixation durations prior to a forward return saccade with those prior to a forward saccade that skipped passed a previously fixated word (i.e., all cases followed an interword regression). We examined this comparison for both intraline fixations and those following undersweep-fixations. Note that the proportion of forward return saccades (FRS) to forward skip saccades (FSS) were nearly identical for intra-line and undersweep cases in each data set. Moreover, on nearly 70% of the undersweep cases, following the corrective saccade, the eyes either immediately returned to, or skipped passed the location (i.e. word) of the undersweep-fixation. Analyses indicated that fixations prior to a skip of a previously fixated word were shorter than those which returned to a previously fixated word, consistent with an IoR effect. Additionally, in the Provo Corpus, fixations prior to returning to or skipping a word that had just received an undersweep-fixation were longer than for intra-line cases. Crucially, both datasets yielded a significant interaction (see Fig. 2) whereby the increased duration of a return saccade relative to a skip saccade was smaller for undersweep cases than for forward return saccades.^3^ Therefore, while there is evidence that IoR may play a role in the subsequent eye-movement behaviour for words that received an undersweep-fixation, these IoR effects are statistically smaller than those for intraline reading.

## Discussion

The current work explored the impact that undersweep-fixations have during online linguistic processing of multiline texts. We asked three specific questions. First, we wanted to know if the durations of undersweep-fixations were influenced by the lexical characteristics of the words they land on. Effects of variables such as word frequency are traditionally viewed as evidence of lexical processing influencing eye-movement behaviour (Rayner, 1998, 2009). Despite strong and significant effects of lexical variables on subsequent skipping and gaze durations in both data sets, neither the Provo Corpus nor our reading comprehension passages showed any evidence of such lexical effects on undersweep-fixation durations themselves. This suggests that the signal to move the eyes on from these fixations does not result from linguistic processing. However, this does not rule out the possibility that processing useful for reading occurs during the fixations.

This possibility was explored in our second question, by examining the subsequent reading of the line for evidence that processing occurred during the earlier undersweep-fixations. We reasoned that during the left to right pass of the lines, skipping rates should be higher and the gaze durations lower for words that had received an undersweep-fixation than for words which did not. Even after controlling for the main effects of lexical variables, there was a significant effect of undersweep-fixations consistent with our prediction (i.e., higher skipping rates and lower gaze durations). These effects, which were present in both data sets that we examined, suggest that something useful for reading is extracted about words during the undersweep-fixations that land on them.

Our third research question explored the possibility that at least a portion of the undersweep preprocessing benefit was due to IoR effects. While it may be apparent how an inhibition of return effect may result in greater skipping of a word that had just been fixated, it may be less obvious to see how IoR could result in shorter subsequent gaze durations. Longer fixations prior to a return saccade could allow for greater parafoveal preview of words that had just received an undersweep-fixation. Examining both intraline and undersweep cases, we found evidence of IoR with the effects being statistically smaller after undersweeps. However, it is difficult to ascertain from the current evidence how much of the undersweep preprocessing benefit effects may be due to IoR, given the lack of an appropriate baseline for reference. Future research is needed to address this question. However, the fact that IoR was detected after undersweep-fixations suggests that attention was at least temporarily at the location of oculomotor error rather than at the intended target of the saccade. This is of importance as E-Z Reader allows for oculomotor error to result in the mislocation of fixations (Drieghe et al., 2007) but not the mislocation of attention.

While there are currently no models of eye movements during reading which simulate return sweeps, the findings reported here will be of great value to constrain future models. Furthermore, the undersweep preprocessing benefit may be difficult for serial attention shift models such as E-Z Reader to account for. While E-Z Reader allows for words to be fixated out of their canonical order, attention for lexical processing follows a strictly serial path. Therefore, according to the model, if the second or more word on a line receives an undersweep-fixation, it would not receive any lexical processing during that fixation. As such, it may be easier for a parallel processing architecture like SWIFT to account for the undersweep preprocessing benefits if it were to be extended to account for return sweeps. However, one potential explanation for the current findings that would be consistent with E-Z Reader, is that the information extracted during an undersweep-fixation is prelexical. That is, it consists of abstract letter identities obtained during the preattentive visual processing stage (V). Representations of abstract letter identities are capable of surviving the masking effects that occur across fixations (McConkie & Zola, 1979). Further research is needed to determine the nature of the information extracted during undersweep-fixations.

## Conclusions

As the field of reading eye-movement research moves toward an understanding of multiline reading, more studies will be confronted with the issue of undersweep-fixations. The current work highlights the impact these fixations can have. Simply deleting these fixations is not sufficient for removing the influence they have on subsequent eye-movement behaviour. Undersweep-fixations not only allow for preprocessing of the words that they land on but also provide significant parafoveal preview benefit of line initial words (Parker & Slattery, 2019). Instead of deleting these fixations, they should be taken into account within statistical analyses. Better still will be a more complete theoretical understanding of the role these fixations play in the ongoing linguistic processing that occurs during natural reading. As researchers continue to tackle the challenges of multiline reading experiments, and modelers begin to incorporate return sweeps into their simulations, such an understanding will develop.

## Footnotes

1 We would like to thank Dr Sarah White for pointing elucidating this argument during her review of an earlier version of this article.

2 Note that the comprehension items data set contained far more forward return saccades than the “Provo Corpus data set. We believe this may have been the result of the large number of comprehension questions (*n* = 10) asked after each of the comprehension items. This likely caused readers to adopt a more cautious reading strategy which included higher regression rates.

3 Follow-up LMMs confirmed that the fixations prior to a forward-skip saccade which takes the eye back passed the word which received an undersweep-fixation were shorter than fixations prior to a saccade that returns to this word. Comprehension data set, *b* = −.025, SE = .012, *t* = −2.12; Provo data set, *b* = −.021, *SE* = .005, *t* = −4.54.
